# Editorial to the Special Issue “Lipidomics and Neurodegenerative Diseases”

**DOI:** 10.3390/ijms22031270

**Published:** 2021-01-28

**Authors:** Cosima Damiana Calvano, Ilario Losito, Tommaso Cataldi

**Affiliations:** 1 Dipartimento di Farmacia-Scienze del Farmaco, Università degli Studi di Bari Aldo Moro, via Orabona 4, 70126 Bari, Italy; 2 Centro Interdipartimentale SMART, Università degli Studi di Bari Aldo Moro, via Orabona 4, 70126 Bari, Italy; ilario.losito@uniba.it (I.L.); tommaso.cataldi@uniba.it (T.C.); 3 Dipartimento di Chimica, Università degli Studi di Bari Aldo Moro, via Orabona 4, 70126 Bari, Italy

**Keywords:** lipidomics, neurodegenerative diseases, sphingolipid, mass spectrometry, biomarker

## Abstract

The contribution of dysregulation of lipid signaling and metabolism to neurodegenerative diseases including Alzheimer’s and Parkinson’s is the focus of this special issue. Here, the matter of three reviews and one research article is summarized.

Lipidomics is a newly emerged discipline that studies cellular lipids of biological systems on a large scale of pathways and networks by exploiting advanced technological tools [1,2], whereby mass spectrometry (MS) is principally involved [3]. Lipids play many essential roles in cellular functions, including energy storage and structural integrity of membranes, cellular homeostasis, regulation of membrane trafficking, and signal transduction as well [4]. Conceivably, dysregulation of lipid signaling and metabolism is generally recognized to contribute to many diseases such as “metabolic syndrome”, which includes hypertension, diabetes, obesity, and atherosclerosis; cancer [5]; and neurological disorders including Alzheimer’s, Parkinson’s, amyotrophic lateral sclerosis, Huntington’s, and multiple sclerosis [6,7]. This Special Issue (SI) entitled “Lipidomics and Neurodegenerative Diseases” focuses its attention on the complexity of the neurolipidome and aims to expand our knowledge of its relationship with neurodegenerative diseases. This SI consists of three reviews and one research article that substantially contribute to the mission of the International Journal of Molecular Sciences, that is, to expand the information available to scientists and readers on molecular studies in systems biology and chemistry.

In the first review [8], Mesa-Herrera et al. describe the complexity of brain lipid composition, the documented lipid changes with aging, and the formation of membrane microdomains named lipid rafts and their functions in neurodegenerative diseases with the aim to discover novel biomarkers. Lipids are mainly abundant in the brain, where they are involved in specific structural functions such as neurogenesis, neural communication, signal transduction, synaptic transmission, membrane compartmentalization, and regulation of gene expression. In nerve cells, lipids represent 50–60% of cell membranes, with cholesterol, glycerophospholipids (GPs), and sphingolipids (SLs) being the most represented in the central nervous system (CNS) [9]. Fatty acyl chains of phospholipids, cholesterol esters, and triglycerides are also significant structural components of neuronal membranes with about 50% of polyunsaturated fatty acids (PUFAs). The lipid portions embedded in the plasma membrane may be fused with proteins generating highly organized multimolecular structures called lipid rafts, which are liquid-ordered domains rich in sphingolipids, phosphatidylcholine, and cholesterol [10]. It is documented that a progressive loss of a certain class of lipids in the brain lipid matrix occurs in humans more than 50 years old, thus suggesting that lipid homeostasis and metabolism are intimately correlated with human brain aging [11]. Considering that the human brain accomplishes a wide range of regulatory, sensory, motor, and cognitive functions which decay with progressing aging, it is reasonable to envisage that regional alterations in the brain lipid medium are responsible for the pathophysiological processes involved in neurodegenerative diseases such as Alzheimer’s disease (AD) and Parkinson’s disease (PD). Indeed, part of these aberrant changes have been related to lipid-raft-integrated proteins, with alterations observed not only in their molecular lipid composition but also in raft organization, changing the local microenvironment with consequences in their physicochemical properties and neuronal impairment. These alterations and their role in promoting neurodegenerative processes were successfully reviewed by Mesa-Herrera et al. [8]. The authors focused also on the fluctuating targeted lipid species as biomarkers for AD, PD, and other synucleinopathies. Table 1 summarizes changes observed in the human brain and peripheral tissues such as cerebrospinal fluid (CSF), plasma, and blood during AD and PD development. Specifically, whereas ceramide and free palmitic and stearic acids seem to be increased, sphingomyelin, GPs, free oleic and linolenic acids, gangliosides, cerebrosides, and sulfatides are found to decrease in AD. Partially overlapping similar trends are also reported for PD, but differences are pointed out [8].

In the second review [12], Rana et al. analyzed how AD and PD could relate to each other from the genomic, epigenomic, and transcriptomic viewpoints since their relationship is still unidentified. AD is clinically characterized by a steady loss of memory and impairment of other cognitive functions such as communication, language inability, advanced visual processing, and movement [13]. The onset of AD is chiefly ascribed to the extracellular β-amyloid (Ab42/40) aggregates and intracellular hyperphosphorylated Tau protein that accumulate in the brain of AD patients, triggering neuroinflammation and brain cell death. PD is clinically characterized by progressive debilitation in motor functions such as resting tremor, rigidity, bradykinesia, gait impairment, and postural instability [14]. PD is caused by the death of dopamine-generating cells of *substantia nigra* in the midbrain region and aggregation of the α-synuclein protein, which disturbs the function of the CNS. Despite occurring at different brain locations and reporting different clinical features, AD and PD share pathological intersections so that patients with AD prove in some cases a higher chance of developing PD. While the mutual pathological overlap may be linked to genes, mitochondrial dysfunction, neuroinflammation, tau protein, and α-synuclein protein [15], common risk factors are signified by oxidative stress, insufficiency of vitamin D, and aging [16]. In the present review [12], Rana et al. first accumulated a list of genes from major genome-wide association (GWAS) studies by recurring to extensive literature mining. As a result of GWAS studies, the authors recovered only one gene (i.e., HLA-DRB5) shared between AD and PD that has been reported earlier several times for AD and PD, exhibiting a strong relationship with the CNS. Previous literature identified a few other common genes, among which SIRT1 looks to be the most notable since it plays a dual role in impacting Ab plaque formation and α-synuclein aggregation. Further, the paper analyzed 15 common miRNAs reported for both diseases that are mainly involved in numerous signaling pathways. The examination of miRNAs using the TAM tool (http://www.lirmed.com/tam2/) [17] with the upregulation option has led to the observation that six miRNAs and five miRNAs are associated with AD and PD, respectively. Apparently, four miRNAs, namely, hsa-miR-181a, hsa-miR-29a, hsa-miR-29b, and hsa-miR-29c, are shared with both diseases, while has-miR-29a and has-miR-16 regulate a common pathway associated with AD and PD. At the end, the authors predicted the gene co-expression system using network analysis algorithms applied to two Gene Expression Omnibus datasets. The network analysis revealed six clusters of genes related to AD and four clusters of genes related to PD with very low functional similarity, suggesting that very different biological processes are activated in both diseases. Perhaps, AD and PD have different genetic roots but converge to a similar phenotypic outcome sharing a few similar symptoms; further, the 15 common miRNAs may serve as a defense mechanism against brain toxicity and may not play a connecting role in either AD or PD.

The third review [18] focuses on a specific lipid class, namely, sphingolipids (SLs), relating their alterations in biofluids, including serum, plasma, and CSF, to several neurodegenerative diseases with the aim of finding diagnostic or prognostic biomarkers. Typically, SLs are localized in the outer leaflet of the plasma bilayer membrane of eukaryotes and some prokaryotes and are supposed to play an important role in transmembrane signaling, cell proliferation and migration, apoptosis, differentiation, senescence, and inflammatory responses [19]. This is especially true in nervous cells, where they are also involved in neuron–glia interactions, synaptic stability, and transmission [20]. First, the chemical structures of sphingoid bases and sphingolipids, more commonly measured in biofluids together with enzymes involved in the SL synthesis and metabolism pathways in different cellular compartments, are briefly described [21]. A focus on the six mammalian ceramide synthases (CerS) and their specificity on acyl chains is given in Figure 2 and Table 1. Then, Section 3 reports lipidomic studies in biofluids from patients with neurodegenerative diseases with a case–control design, while Section 4 focuses on longitudinal studies as a tool to find prognosticator lipids of a phenotypic feature or disease state. Case–control studies have been carried out in several neurodegenerative diseases such as AD, PD, multiple sclerosis (MS), dementia with Lewy bodies (DLB), and age-related macular degeneration (AMD) and from different biofluids such as blood, CSF, and plasma, measuring both ceramides and sphingomyelins, or focusing on one lipid class. A common alteration in the investigated neurodegenerative disorders is the relative content increase of Cer d18:1/16:0 in patients compared with controls (see Table 2 and Figure 3); other ceramides were found steadily increased in biofluids of AD patients and hexosylceramide species in plasma of PD patients. On the contrary, the sphingomyelins (SM) followed different patterns (Table 3), with a content decrease in neurodegenerative diseases compared to controls except for AMD, where an increase in SM 42:2 and 42:3 was reported [22]. The longitudinal studies account for some contrasting results regarding SL variation between women and men, but in men, higher levels of Cer d18:1/16:0, 18:0, 22:0, and 24:0 and SM d18:1/18:0, 18:1, 20:1, and 22:1 were correlated with the increased risk of developing AD in agreement with case–control studies.

These changes of SL levels observed in biofluids may reflect alterations in nervous degenerating tissues, so a correlation has been inspected by comparing studies carried out on white matter of temporal cortex and cerebellum, prefrontal cortex, frontal gyrus, and anterior cingulate cortex. Again, in brain tissues, an increased content of Cer d18:1/16:0 is described in agreement with most of the case–control and longitudinal studies on biofluids. Indeed, the recurrent altered level of Cer C16:0 has been associated with its capacity to mix well with cholesterol and consequently influence the functionality of lipid rafts that, becoming enriched in ceramide, activate both intrinsic and extrinsic apoptotic pathways [23]. Lipid micelles containing Cer C16:0 induced oxidative stress and decreased neuronal maximal respiratory rate and capacity in primary rat hippocampal neurons, apoptosis in neutrophils, and apoptosis in rodent hepatocytes. Thus, the shifts in the SL composition and expression observed in neurodegeneration could be associated with regulation of CerS or sphingomyelinases (SMase). The review of Pujol-Lereis [18] highlights that plasma-altered lipids and genetic variants could be correlated, but validation in analytical methods and disease-specific SL patterns are needed to translate findings to clinical approaches.

The fourth research paper of this SI [24] aimed to discover neuronal changes occurring in PD patients by investigating human skin fibroblasts as novel tissue/culture peripheral cells. Early diagnosis of neural fluctuation causing cerebral impairment is critical for recommending preventive therapies in PD, and proposed biomarkers are not informative of PD onset since they are obtained from postmortem tissues from patients with advanced degeneration of the *substantia nigra*. Skin fibroblasts are now broadly recognized as a suitable model of primary human cells, able to reflect the chronological and biological aging of patients since they share the genetic information of neuron sprouts [25]. A lipidomics study of easily accessible primary human fibroblasts is presented here based on hydrophilic interaction liquid chromatography coupled to electrospray ionization–Fourier transform mass spectrometry. Phospholipids (PL) from dermal fibroblasts of five PD patients with different parkin mutations and normal human dermal fibroblasts as a control were characterized, and about 360 PL were identified in terms of head groups, backbones, and fatty acyl moieties (SLs in skin fibroblasts are stated in a dedicated work [26]). By univariate analysis, a total of 61 PL were found to be significantly different between the control and PD groups (*p* < 0.05); among them, 8 lipids, namely, PI 38:3, PC 34:1, PE 38:2, and SM 36:2;2, and 4 plasmalogens seem to follow an ascending or descending trend as the severity of the disease increases. Statistical analyses show abnormality of GP metabolism in the PD group, suggesting that the downregulation of ethanolamine plasmalogen (pPE) supply in the circulation system, especially those containing PUFA, might be likely associated with neurodegeneration. However, for a definitive application of these potential biomarkers in clinical diagnosis, validation in a larger population should be further achieved.
